# Proteomic analysis of extracellular vesicles enriched serum associated with future ischemic stroke

**DOI:** 10.1038/s41598-021-03497-0

**Published:** 2021-12-15

**Authors:** Shingo Mitaki, Yasuko Wada, Abdullah Md. Sheikh, Shuhei Yamaguchi, Atsushi Nagai

**Affiliations:** 1 Department of Neurology, Shimane University School of Medicine, 89-1 Enya-cho, Izumo, 693-8501 Japan; 2 Department of Laboratory Medicine, Shimane University School of Medicine, 89-1 Enya-cho, Izumo, 693-8501 Japan; 3 Shimane Prefectural Central Hospital, 4-1-1 Himebara, Izumo, Shimane 693-8555 Japan

**Keywords:** Neuroscience, Biomarkers, Neurology

## Abstract

Identifying new biomarkers beyond the established risk factors that make it possible to predict and prevent ischemic stroke has great significance. Extracellular vesicles are powerful cell‒cell messengers, containing disease-specific biomolecules, which makes them powerful diagnostic candidates. Therefore, this study aimed to identify proteins derived from extracellular vesicles enriched serum related to future ischemic stroke events, using a proteomic method. Of Japanese subjects who voluntarily participated in health checkups at our institute a number of times, 10 subjects (6 males and 4 females, age: 64.2 ± 3.9 years) who developed symptomatic ischemic stroke (7.3 ± 4.4 years’ follow-up) and 10 age‒sex matched controls without brain lesions (6.7 ± 2.8 years’ follow-up) were investigated. Extracellular vesicles enriched fractions were derived from serum collected at the baseline visit. Differentially expressed proteins were evaluated using isobaric tagging for relative and absolute protein quantification (iTRAQ)-based proteomic analysis. Of the 29 proteins identified, alpha-2-macroglobulin, complement C1q subcomponent subunit B, complement C1r subcomponent, and histidine-rich glycoprotein were significantly upregulated (2.21-, 2.15-, 2.24-, and 2.16-fold, respectively) in subjects with future ischemic stroke, as compared with controls. Our study supports the concept of serum-derived extracellular vesicles enriched fractions as biomarkers for new-onset stroke. These proteins may be useful for prediction or for targeted therapy.

## Introduction

To date, several risk factors for ischemic stroke (e.g., smoking, atrial fibrillation, hypertension, dyslipidemia, and impaired glucose metabolism) have been established^1^. Despite advances in therapeutic interventions targeting these conventional risk factors, ischemic stroke remains one of the leading causes of death and disability in Japan. Furthermore, due to the rapid aging of the population, the number of stroke patients is expected to increase. In this context, there is a need to identify new biomarkers relevant to ischemic stroke. Identifying new biomarkers will improve our understanding of the pathophysiology of stroke and could suggest novel therapeutic targets, and may facilitate risk prediction.

Extracellular vesicles (EVs) are nano-scale vesicles secreted by multiple cells through endogenous pathways^2^. EVs contain various functional molecules, including lipids, proteins, and nucleic acids, which act as intracellular biological signals from cell to cell or organ^3^. Since EVs-mediated signal transmission plays essential roles in both physiological and pathological conditions^4,5^, EVs have become a focal point in the search for biomarkers of different disorders. Previous studies have suggested that circulating EVs can influence the course of cardiovascular diseases^6,7^. However, no data are available in the general population regarding the value of EVs for prediction of stroke risk.

EVs possess unique features over conventional serological markers and have a higher sensitivity than proteins directly detected in blood, as well as a higher specificity than secretory proteins^8^. Given that EVs may be altered in abundance or physical properties associated with various diseases, they could putatively provide information about disease development.

Therefore, this study aimed to identify serum-derived EVs enriched fractions related to new-onset ischemic stroke, using an iTRAQ-based proteomic method, in a Japanese health examination cohort. iTRAQ has recently been demonstrated to be one of the most robust techniques for quantifying proteins based on peptide labeling and allows for the identification and accurate quantification of proteins from multiple samples, within a broad dynamic range of protein abundance^9,10^.

## Methods

### Study population

From January 2000 to December 2019, 6582 Japanese subjects voluntarily participated in a health checkup at the Shimane Institute of Health Science. Of these, 1093 subjects who participated several times and were followed-up for at least 1 year after the initial examination were included in the present study. Medical, neurological, and psychiatric histories were collected from the subjects. Neurological examinations were performed by an experienced neurologist. Subjects also underwent head magnetic resonance imaging (MRI), and blood tests. To confirm whether subjects developed ischemic stroke during the study period, questionnaires were mailed to all subjects on an annual basis. Of 1093 subjects, 10 subjects (6 males and 4 females, mean age: 64.2 ± 3.9 years) who developed symptomatic ischemic stroke (classified as the future ischemic stroke group, mean 7.3 ± 4.4 years’ follow-up) and randomly selected 10 age- and sex-matched controls, without development of any brain lesions (classified as the control group, mean 6.7 ± 2.8 years’ follow-up) were investigated.

Our study was approved by the Ethics Committee of the Shimane University School of Medicine in Japan, and all participants provided written informed consent in accordance with the Declaration of Helsinki. The procedures followed were in accordance with institutional guidelines.

### Blood collection and assessment of clinical variables

At the first visit, after overnight fasting, blood samples were drawn into tubes containing silica particles and an inert gel separator for serum preparation, and tubes containing sodium fluoride and EDTA for plasma preparation. Subsequently, the blood samples were immediately centrifuged at 3000 rpm × 5 min at room temperature, and serum total cholesterol (TC), high-density lipoprotein (HDL-C), low-density lipoprotein cholesterol (LDL-C), triglyceride (TG), fasting plasma glucose, creatinine (Cr), and hemoglobin A_1_C levels were measured. Simultaneously, serum samples were stored at − 80 °C for isolation of EVs enriched fractions.

Hypertension was defined as systolic/diastolic blood pressure ≥ 140/90 mmHg or treatment with antihypertensive drugs. Diabetes mellitus was defined as a fasting blood glucose level ≥ 126 mg/dL, a random blood glucose level ≥ 200 mg/dL, or treatment with oral antidiabetic drugs or insulin. Hyperlipidemia was defined as a serum cholesterol level ≥ 220 mg/dL or treatment with lipid-lowering drugs. The estimated glomerular filtration rate (eGFR) was calculated using the following formula: eGFR (mL/min/1.73 m^2^) = 194 × Cr^−1.094^ × age^−0.287^ for men and 194 × Cr^−1.094^ × age^−0.287^ × 0.739 for women. Smoking and drinking status were defined as follows: a smoker was a person who habitually smoked at least 1 cigarette per day and a drinker was a person who consumed ≥ 180 mL of alcohol per day.

### Imaging data

To define a control group without development of any brain lesions, head MRI was performed at the first and last visits. Brain infarction was defined as a focal hyperintense lesion ≥ 3 mm in diameter on the T2-weighted images. Cerebral microbleeds were defined as homogenous 2‒10-mm round foci of signal loss on gradient-echo T2*-weighted images. Periventricular hyperintensities and white matter hyperintensities were defined as hyperintense lesions in fluid-attenuated inversion recovery images. All MRI findings were read and determined separately by an experienced neurologist and a radiologist who was blinded to the patients’ profiles.

### Isolation of EVs enriched fractions and protein extraction

EVs enriched fractions were isolated from serum at the first visit using the Exo Trap™ exosome isolation spin column kit (Cosmo Bio, Tokyo, Japan) following the manufacturer’s instructions. Briefly, 500 µL of serum was diluted three times with PBS and centrifuged at 12,000×*g* for 4 min. The supernatant was filtered through a 0.22-μm disc filter (STRALAB, Milton Keynes, UK) and was applied to the Exo Trap™ column and then centrifuged at 5000×*g* for 1 min. After washing with phosphate-buffered saline (PBS), exosomal protein from the column was eluted with urea lysis buffer (7 M urea, 0.1% Nonidet P-40, and 500 mM triethylammonium bicarbonate) for iTRAQ-based quantitative proteomics and with RIPA buffer (PBS, pH 7.4, 1% Nonidet P-40, 0.5% sodium deoxycholate, 0.1% SDS, 10 mg/mL PMSF, and 1 mg/mL aprotinin) for enzyme-linked immunosorbent assay (ELISA). Protein concentration was determined using a bicinchoninic acid assay kit (Thermo Fisher Scientific, Waltham, MA, USA).

### Antibody array

The immunoblotting analyses of the EVs-specific markers were performed to validate the separated EVs using a commercial Exo-Check™ Exosome Antibody Array (Neuro) Mini (System Biosciences, Palo Alto, CA, USA). The same amount of EVs enriched fractions (30 μg) was added to the membrane-based blot array according to the manufacturer’s instructions. Briefly, the EVs enriched fractions were lysed by a lysis buffer, and labelled using the labelling reagent. Following incubation for 30 min, the labelled sample was washed through the column and blocked by 5 mL blocking buffer. The membrane was incubated with 5 mL labelled EVs lysate/blocking buffer mixture at 4 °C overnight with shaking. Next day, the membrane was washed with a wash buffer and incubated using a detection buffer followed by development with enhanced chemiluminescence (ECL; Cytiva, Tokyo, Japan); the chemiluminescent signals were captured with the Amersham ImageQuant 800 (Cytiva, Tokyo, Japan).

### ELISA

To evaluate the extraction efficiency of EVs enriched fractions between the two groups, the absolute expression levels of CD9 were measured in all samples from each group, using a commercial ELISA kit (CUSABIO, Wuhan, China) according to the manufacturer’s instructions. Briefly, the EVs enriched fractions were diluted with sample diluent, and 25 μL of the sample or standards were added per well. After the reaction, the optical density of each well was determined immediately, using a microplate reader set to 450 nm.

### iTRAQ labeling and strong cationic exchange fractionation

To minimize biological variation, 10 samples were pooled in each group. Samples from the future ischemic stroke group were labeled with 114 iTRAQ labels, and samples from the control group were labeled with 115 iTRAQ labels. iTRAQ labeling and strong cationic exchange fractionation were performed as previously described^11^.

### Nano liquid chromatography and mass spectrometry analysis

Peptide fractions were first separated by nano liquid chromatography (LC; DiNa nanoLC system; KYA Tech, Tokyo, Japan) as described previously^11^. Then, fractionated peptides were analyzed on a MALDI time-of-flight (TOF)/TOF 5800 MS/MS Analyzer with TOF/TOF Series software (version 4.0) (AB Sciex). The MS was performed in positive ion mode across the mass range of 800–4000 m/z. Duplicate LC–MS/MS runs per fractions were performed.

### Database research

For protein identification, the Paragon algorithm^12^, which was integrated with Protein Pilot 3.0 software, was analyzed using the UniProt/International Protein Index protein sequence database for Human. Proteins were identified according to the following criteria: i) a false discovery rate (FDR) < 5% (FDR was estimated by “decoy database searching” using Protein Pilot software); and ii) protein confidence > 95% (“unused ProtScore” > 1.3). The Unused ProtScore was defined as − log (1 − % confidence/100). Comparisons of differential protein expression in the two groups were made with the statistical significance level set at p < 0.05, and a 1.2-fold change (> 1.20, or < 0.83) threshold.

### Statistical analysis

Clinical characteristics of study participants were compared between the two groups using Student’s *t*-test or Fisher’s exact test. The expression levels of CD9 obtained by ELISA were compared using Student’s *t* test. All analyses were performed using SPSS version 23 (IBM Corp., Armonk, NY, USA). Statistical significance was set at p < 0.05.

To confirm that the sample size was large enough to detect significant differences, a post-hoc power analysis was conducted using G ∗ Power v3.1.9.

### Ethics approval

All procedures performed in studies involving human participants were in accordance with the ethical standards of the institutional and national research committee and with the 1964 Helsinki Declaration and its later amendments or comparable ethical standards. The study was approved by the Bioethics Committee of the Shimane University (No. 20160217-1).

## Results

### Baseline characteristics

The baseline characteristics of the study participants are shown in Table 1. Subjects with future ischemic stroke were more likely to have hypertension (80% vs. 30%; P = 0.03); however, there were no significant differences in other clinical characteristics. The differences of baseline characteristics between included and excluded subjects are also shown in supplementary table1. Included subjects are older and the proportion of female are lower than those in excluded subjects, however, no significant differences are seen in other characteristics.

**Table 1:** Baseline characteristics of study population.

	Future ischemic stroke	Control	p
Age (SD), years	64.2 (3.9)	64.4 (4.9)	ns
Sex, female %	40	40	ns
Follow-up (SD), years	7.3 (4.4)	6.7 (2.8)	ns
Education (SD), years	12.7 (3.1)	13.9 (2.6)	ns
Hypertension, %	80	30	0.03
Systolic BP (SD), mmHg	138.1 (14.2)	126.3 (16.0)	ns
Diastolic BP (SD), mmHg	78.2 (9.8)	74.0 (10.2)	ns
Hyperlipidemia, %	30	60	ns
Total cholesterol (SD), mg/dL	210 (21.4)	208 (36.1)	ns
HDL cholesterol (SD), mg/dL	61.7 (10.5)	67.9 (16.6)	ns
LDL cholesterol (SD), mg/dL	128.3 (17.6)	119.8 (34.1)	ns
Triglyceride (SD), mg/dL	105 (38.9)	106.3 (44.4)	ns
Diabetes mellitus, %	10	0	ns
Blood glucose (SD), mg/dL	102.6 (10.7)	97.3 (8.6)	ns
Hemoglobin A1c (SD), %	5.3 (0.5)	5.4 (0.2)	ns
eGFR (SD), mL/min/1.73 m^2^	66.9 (11.9)	74.3 (13.3)	ns
Atrial fibrillation, %	0	0	ns
Smoking, %	10	0	ns
Drinking, %	20	30	ns

### Proteomic profiles of EVs enriched fractions

**Figure 1:**
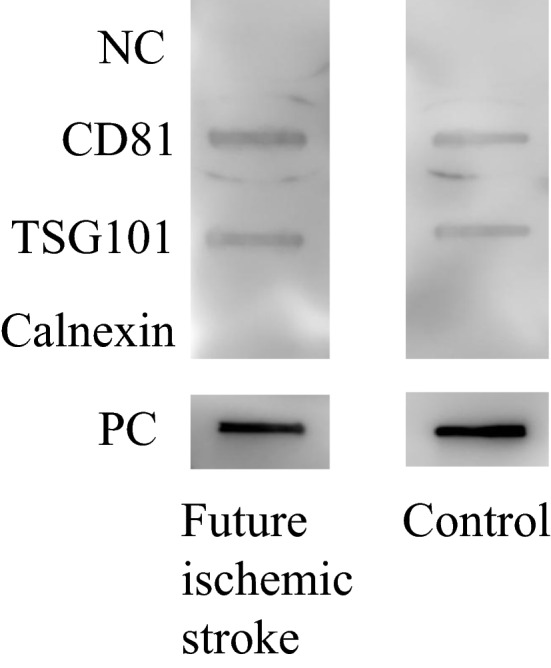
EVs marker expression analysed by immunoblotting. *NC* negative control, *PC* positive control.

**Figure 2:**
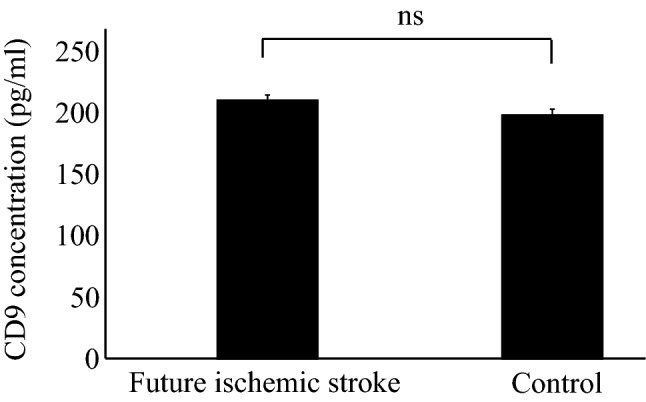
CD9 concentrations in subjects with future ischemic stroke and controls, analyzed by enzyme-linked immunosorbent assay.

First, the expression of the EVs marker proteins, CD81 and TSG101, were evaluated using immunoblotting. As shown in Fig. 1, CD81 and TSG101 were expressed in both the samples, while calnexin, which generally represents contamination by intracellular proteins, was absent. To evaluate extraction efficiency of EVs enriched fractions, the concentration of CD9 was determined by ELISA. As shown in Fig. 2, there was no significant difference in the CD9 concentration between the two groups. The run-to-run technical variation was then determined based on the percentage coefficient variation (%CV). The overall %CV accomplished by the duplicate measurements was 9.96% for identified proteins, 9.75% for peptides, and 9.21% for spectra. This confirmed minimal variation and sufficient reproducibility.

Then, a combined duplicate LC–MS/MS database search was performed. Based on the criteria described above, we identified 29 quantified proteins (Table 2). Of the 29 proteins detected, potential biomarkers of future ischemic stroke were identified using the iTRAQ ratios 114_future ischemic stroke_:115_control_. The initial list of 29 proteins included four potential biomarkers that exhibited significant differential expression between the two groups. As shown in Table 2, alpha-2-macroglobulin (A2MG), complement C1q subcomponent subunit B (C1QB), complement C1r subcomponent (C1R), and histidine-rich glycoprotein (HRG) were significantly upregulated by 2.21-, 2.15-, 2.24-, and 2.16-fold, respectively, in the ischemic stroke group as compared to the control group.

**Table 2:** Quantitative information of proteins associated with future ischemic stroke.

Protein name	Uniprot ID	Unused	Peptide (95%)	Protein coverage (95%)	Ratio (114/115)	p
Alpha-1-antitrypsin	P01009	4.6	3	4.1	1.05	ns
Alpha-2-antiplasmin	P08697	2.3	1	1.8	0.94	ns
Alpha-2-HS-glycoprotein	P02765	6	3	6.3	2.08	ns
Alpha-2-macroglobulin	P01023	10.5	4	2.9	2.21	0.002
Apolipoprotein A-I	P02647	26.6	14	37.1	1.24	ns
Apolipoprotein B-100	P04114	12.9	6	1.5	1.04	ns
CD5 antigen-like	O43866	3.7	2	4.6	0.69	ns
Complement C1q subcomponent subunit A	P02745	8.9	7	24.9	0.93	ns
Complement C1q subcomponent subunit B	P02746	6.0	12	11.9	2.15	0.045
Complement C1q subcomponent subunit C	P02747	8.1	9	9.8	3.28	ns
Complement C1r subcomponent	P00736	18.0	12	8.5	2.24	0.006
Complement C1s subcomponent	P09871	12.0	6	9.0	1.24	ns
Complement C3	P01024	6.3	3	2.4	0.94	ns
Complement C4-B	P0C0L5	3.4	1	0.4	1.91	ns
Complement factor H	P08603	3.2	1	0.9	1.31	ns
Galectin-3-binding protein	Q08380	10.1	6	8.2	1.71	ns
Haptoglobin	P00738	10.2	5	15.0	1.04	ns
Histidine-rich glycoprotein	P04196	38.0	34	28.8	2.16	0.01
Immunoglobulin gamma-1 heavy chain	P0DOX5	11.1	19	27.4	0.83	ns
Immunoglobulin heavy constant gamma 3	P01860	34.4	24	38.5	1.90	ns
Immunoglobulin heavy constant mu	P01871	34.1	29	35.5	0.59	ns
Immunoglobulin kappa light chain	P0DOX7	14.5	19	26.2	0.94	ns
Immunoglobulin lambda constant 3	P0DOY3	5.4	9	48.1	0.99	ns
Inter-alpha-trypsin inhibitor heavy chain H4	Q14624	4.3	2	2.2	1.05	ns
Kininogen-1	P01042	8	6	6.5	2.20	ns
Plasminogen	P00747	3.1	1	0.9	2.19	ns
Serotransferrin	P02787	10.6	6	5.2	1.14	ns
Serum albumin	P02768	44.5	30	34.0	1.18	ns
Vitronectin	P04004	4.5	2	4.0	1.10	ns

Finally, statistical power was calculated using linear regression model with α = 0.05 which represent the common choices for significance and power analyses. The effect size was calculated as log_10_(fold change) for each protein^13^. Statistical power for A2MG, C1QB, C1R, and HRG were 0.81, 0.80, 0.82, and 0.80 respectively, indicating that the sample size was adequate in this study.

## Discussion

In this study, we characterized the altered protein expression profiles of EVs enriched fractions isolated from the serum of subjects with ischemic stroke and of healthy controls using iTRAQ quantitative proteomics. We revealed marked upregulation of four proteins (A2MG, C1QB, C1R, and HRG) in EVs enriched fractions by 7.3 ± 4.4 years before ischemic stroke onset, indicating their potential as biomarkers for predicting future ischemic stroke events.

A2MG is a glycoprotein of approximately 720 kDa that functions as a broad-spectrum proteinase inhibitor^14^. Since the blood fibrinolytic system consists of a serine proteinase cascade, A2MG might be related to future stroke by altering this cascade. Previously, it was reported that A2MG inhibits plasmin, plasminogen activator, and metalloproteinases^15–18^, all of which are essential for the fibrinolytic cascade. In addition, A2MG inhibits activated protein C, one of the most important anticoagulant proteins classified as a serine protease. In vitro studies have shown that A2MG inhibits activated protein C in a dose-dependent manner^19^. Indeed, it has been reported that elevated A2MG independently increases the risk of thrombosis, including stroke and deep-vein thrombosis^20^. Similarly, HRG, a glycoprotein of approximately 70 kDa, has been reported to modulate the blood fibrinolytic system^21^. Previously, it has been reported that HRG inhibits fibrinolysis by forming a complex with tissue plasminogen activator and plasminogen^22^. Given that nearly 50% of plasminogen circulates in a form bound to HRG^23^, upregulation of HRG serves to reduce the effective plasminogen concentration, thus resulting in retarded fibrinolysis. A previous report that elevated plasma HRG levels are related to thrombophilia^24^ also supports our findings. Taken together, by shifting blood to a hypofibrinolytic state, both A2MG and HRG may be involved in future ischemic stroke.

Interestingly, C1R, C1QB, A2MG, and HRG all belong to inflammatory and immune-related molecules. It is now well established that atherosclerosis is an inflammatory disease^25^. Considering that C1R and C1QB are part of the C1 complex that initiates the classical pathway of the complement cascade, our results indicate that an abnormal complement status might be involved in the development of ischemic stroke. Activation of the complement system is a major aspect of many chronic inflammatory diseases^26,27^, such as the initiation and progression of atherosclerosis^25,28^. Although the complement system is thought to have protective effects in the local environment, such as atherosclerotic plaques on the arterial wall^29^, higher levels of C1Q or C1rC1sC1inh (activation products of the classical pathway) in systemic circulation have been associated with poor cardiovascular outcomes^30,31^, which was consistent with our results. On the other hand, A2MG reportedly behaves as a carrier protein that binds with some proinflammatory cytokines, such as IL-1 beta, IL-6, and TNF-alpha, to harmonize the immune system^32,33^. Similarly, HRG has been reported to bind C1Q, inhibiting the formation of insoluble immune complexes^34^. Based on these reports, upregulation of A2MG and HRG could arise from synchronization with the activated complement system. In terms of chronic inflammation, both A2MG and HRG might merely be indicators of future ischemic stroke and have little direct or causal effects on the pathogenesis of disease development.

This study had several limitations. First, the sample size was small. Second, subjects in our study were recruited from health examination cohort, which may not be generalized to other population. Third, the diagnosis of ischemic stroke was based on questionnaires; therefore, we cannot rule out measurement errors. Thus, bias stemmed from the self-reporting design should be take into consideration. Further studies using diagnostic procedures based on hospital or medical staff are warranted. Fourth, although EVs markers were detected in immunoblotting, there is no optimized and universally-accepted protocol for isolating pure EVs, and contamination of EVs with serum proteins represents an inherent limitation in studies with EVs. Hence, we have described ‘EVs enriched fractions’ instead of EVs throughout the paper. Fifth, the small amount of the starting serum and low yield of the resulting EVs enriched fractions did not allow for the extensive validation of the candidate makers on several types of the control samples. Finally, detailed information about the subjects’ medications that may have influenced the levels of EVs enriched fractions-derived proteins to some extent were not available.

In conclusion, to the best of our knowledge, no previous study has reported on circulating EVs enriched fractions-derived protein biomarker discovery for predicting the risk of future ischemic stroke. A2MG, C1QB, C1R, and HRG were found to be potential predictors of ischemic stroke development; however, further validation and investigation are necessary to determine the roles of all of these differentially expressed proteins in ischemic stroke development.

## Supplementary Information

Supplementary Information 1.

Supplementary Information 2.

## Data Availability

The data of this study are available from the corresponding author, upon reasonable request. Shingo Mitaki and Atsushi Nagai have full access to all the data in the study and take responsibility for the integrity of the data, the accuracy of the data analysis, and the conduct of the research.

## References

[1] O'Donnell MJ (2010). Risk factors for ischaemic and intracerebral haemorrhagic stroke in 22 countries (the INTERSTROKE study): A case-control study. Lancet.

[2] Kalluri R, LeBleu VS (2020). The biology, function, and biomedical applications of exosomes. Science.

[3] Pegtel DM, Gould SJ (2019). Exosomes. Annu. Rev. Biochem..

[4] Yanez-Mo M (2015). Biological properties of extracellular vesicles and their physiological functions. J. Extracell Vesicles.

[5] Gonzalez E, Falcon-Perez JM (2015). Cell-derived extracellular vesicles as a platform to identify low-invasive disease biomarkers. Expert Rev Mol Diagn.

[6] Loyer X, Vion AC, Tedgui A, Boulanger CM (2014). Microvesicles as cell-cell messengers in cardiovascular diseases. Circ. Res..

[7] Kleinjan A, Boing AN, Sturk A, Nieuwland R (2012). Microparticles in vascular disorders: How tissue factor-exposing vesicles contribute to pathology and physiology. Thromb. Res..

[8] Li A, Zhang T, Zheng M, Liu Y, Chen Z (2017). Exosomal proteins as potential markers of tumor diagnosis. J. Hematol. Oncol..

[9] Wang H, Alvarez S, Hicks LM (2012). Comprehensive comparison of iTRAQ and label-free LC-based quantitative proteomics approaches using two *Chlamydomonas reinhardtii* strains of interest for biofuels engineering. J. Proteome Res..

[10] Wiese S, Reidegeld KA, Meyer HE, Warscheid B (2007). Protein labeling by iTRAQ: A new tool for quantitative mass spectrometry in proteome research. Proteomics.

[11] Mitaki S (2020). iTRAQ-based proteomic analysis after mesenchymal stem cell line transplantation for ischemic stroke. Brain Res..

[12] Shilov IV (2007). The Paragon Algorithm, a next generation search engine that uses sequence temperature values and feature probabilities to identify peptides from tandem mass spectra. Mol. Cell Proteom..

[13] Karp NA, Lilley KS (2005). Maximising sensitivity for detecting changes in protein expression: Experimental design using minimal CyDyes. Proteomics.

[14] Cater JH, Wilson MR, Wyatt AR (2019). Alpha-2-macroglobulin, a hypochlorite-regulated chaperone and immune system modulator. Oxid. Med. Cell Longev..

[15] Baugh RF, Thomson JM (1980). Blood Coagulation and Haemostasis: A Practical Guide.

[16] Hermogenes AL (2006). Interaction of a plasminogen activator proteinase, LV-PA with human alpha2-macroglobulin. Toxicon.

[17] Baker AH, Edwards DR, Murphy G (2002). Metalloproteinase inhibitors: Biological actions and therapeutic opportunities. J. Cell Sci..

[18] Tortorella MD (2004). Alpha2-macroglobulin is a novel substrate for ADAMTS-4 and ADAMTS-5 and represents an endogenous inhibitor of these enzymes. J. Biol. Chem..

[19] Cvirn G, Gallistl S, Muntean W (2001). Alpha-2-macroglobulin inhibits the anticoagulant action of activated protein C in cord and adult plasma. Haemostasis.

[20] Beheiri A (2007). Role of elevated alpha2-macroglobulin revisited: Results of a case-control study in children with symptomatic thromboembolism. J. Thromb. Haemost..

[21] Jones AL, Hulett MD, Parish CR (2005). Histidine-rich glycoprotein: A novel adaptor protein in plasma that modulates the immune, vascular and coagulation systems. Immunol. Cell Biol..

[22] Lijnen HR, Hoylaerts M, Collen D (1980). Isolation and characterization of a human plasma protein with affinity for the lysine binding sites in plasminogen. Role in the regulation of fibrinolysis and identification as histidine-rich glycoprotein. J. Biol. Chem..

[23] Wakabayashi S, Koide T (2011). Histidine-rich glycoprotein: A possible modulator of coagulation and fibrinolysis. Semin. Thromb. Hemost..

[24] Engesser L, Kluft C, Briet E, Brommer EJ (1987). Familial elevation of plasma histidine-rich glycoprotein in a family with thrombophilia. Br. J. Haematol..

[25] Ross R (1999). Atherosclerosis–an inflammatory disease. N. Engl. J. Med..

[26] Walport MJ (2001). Complement. First of two parts. N. Engl. J. Med..

[27] Walport MJ (2001). Complement. Second of two parts. N. Engl. J. Med..

[28] Libby P (2002). Inflammation in atherosclerosis. Nature.

[29] Speidl WS, Kastl SP, Huber K, Wojta J (2011). Complement in atherosclerosis: Friend or foe?. J. Thromb. Haemost..

[30] Hertle E (2018). Classical pathway of complement activation: Longitudinal associations of C1q and C1-INH with cardiovascular outcomes: The CODAM Study (Cohort on Diabetes and Atherosclerosis Maastricht)-brief report. Arterioscler. Thromb. Vasc. Biol..

[31] Horvath Z (2013). Elevated C1rC1sC1inh levels independently predict atherosclerotic coronary heart disease. Mol. Immunol..

[32] Gonias SL (2000). Identical or overlapping sequences in the primary structure of human alpha(2)-macroglobulin are responsible for the binding of nerve growth factor-beta, platelet-derived growth factor-BB, and transforming growth factor-beta. J. Biol. Chem..

[33] LaMarre J, Wollenberg GK, Gonias SL, Hayes MA (1991). Cytokine binding and clearance properties of proteinase-activated alpha 2-macroglobulins. Lab. Invest..

[34] Gorgani NN, Parish CR, Easterbrook Smith SB, Altin JG (1997). Histidine-rich glycoprotein binds to human IgG and C1q and inhibits the formation of insoluble immune complexes. Biochemistry.

