# Supplementary material for: Proteomic analysis of extracellular vesicles enriched serum associated with future ischemic stroke
Source: Sci Rep. 2021 Dec 15;11:24024. doi: 10.1038/s41598-021-03497-0 (PMC8674262; doi:10.1038/s41598-021-03497-0)
Supplement: Supplementary file 1 — Supplementary Information 1. [file 41598_2021_3497_MOESM1_ESM.pptx]

## Slide 1
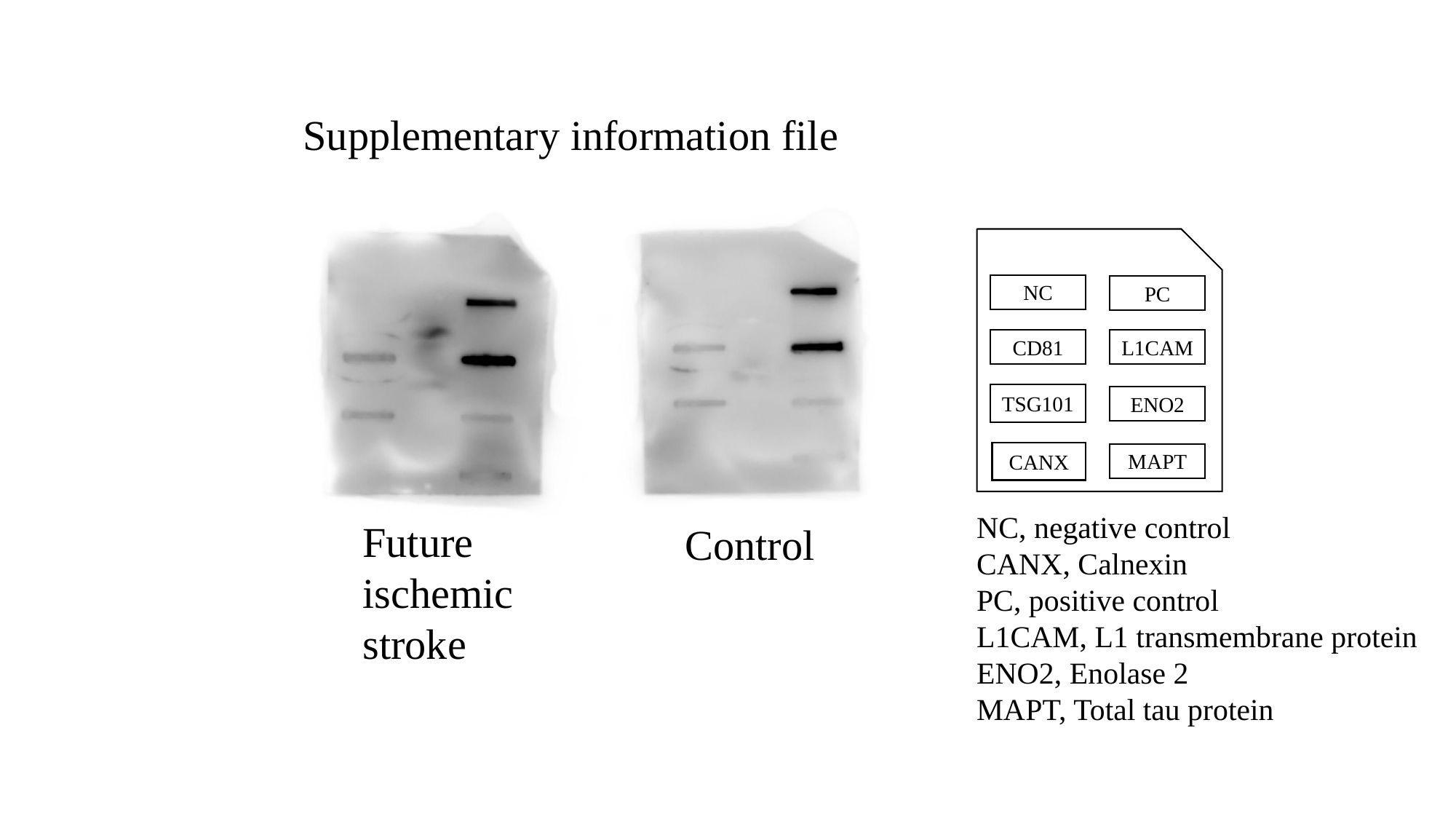

Supplementary information file
NC
PC
L1CAM
CD81
TSG101
ENO2
CANX
MAPT
NC, negative control
CANX, Calnexin
PC, positive control
L1CAM, L1 transmembrane protein
ENO2, Enolase 2
MAPT, Total tau protein
Future
ischemic
stroke
Control
