# Supplementary material for: Proteomic analysis of extracellular vesicles enriched serum associated with future ischemic stroke
Source: Sci Rep. 2021 Dec 15;11:24024. doi: 10.1038/s41598-021-03497-0 (PMC8674262; doi:10.1038/s41598-021-03497-0)
Supplement: Supplementary file 2 — Supplementary Information 2. [file 41598_2021_3497_MOESM2_ESM.docx]

Supplementary table 1

Baseline characteristics of included and excluded subjects

|  | Included subjects | Excluded subjects | p |
| --- | --- | --- | --- |
| Age (SD), years | 60.3 (9.3) | 58.3 (9.9) | <0.0001 |
| Sex, female % | 40.3 | 46.1 | <0.0001 |
| Education (SD), years | 13.3 (5.4) | 13.1 (3.9) | ns |
| Hypertension, % | 37.3 | 34.5 | ns |
| Systolic BP (SD), mmHg | 127.6 (17.2) | 127.0 (17.5) | ns |
| Diastolic BP (SD), mmHg | 73.3 (11.0) | 73.4 (11.4) | ns |
| Hyperlipidemia, % | 43.7 | 46.4 | ns |
| Total cholesterol (SD), mg/dL | 209.3 (32.2) | 212.1 (46.6) | ns |
| HDL cholesterol (SD), mg/dL | 62.8 (16.0) | 63.0 (16.0) | ns |
| LDL cholesterol (SD), mg/dL | 122.4 (30.2) | 123.3 (31.3) | ns |
| Triglyceride (SD), mg/dL | 116.1 (69.6) | 120.2 (82.7) | ns |
| Diabetes mellitus, % | 7.9 | 8.8 | ns |
| Blood glucose (SD), mg/dL | 102.3 (19.9) | 104.1 (24.0) | ns |
| Hemoglobin A1c (SD), % | 5.4 (0.6) | 5.6 (9.3) | ns |
| eGFR (SD), mL/min/1.73 m^2^ | 76.7 (13.1) | 77.3 (14.2) | ns |
| Atrial fibrillation, % | 0.7 | 1.0 | ns |
| Smoking, % | 15 | 16.8 | ns |
| Drinking, % | 21.3 | 21.7 | ns |
